# Comprehensive Analysis Identifies Potential Ferroptosis-Associated mRNA Therapeutic Targets in Ovarian Cancer

**DOI:** 10.3389/fmed.2021.644053

**Published:** 2021-03-05

**Authors:** Jiyan Zhang, Jie Xi, Ping Huang, Saitian Zeng

**Affiliations:** ^1^Department of Gynecologic Oncology, Cangzhou Central Hospital, Cangzhou, China; ^2^Department I of Obstetrics and Gynecology, Cangzhou Central Hospital, Cangzhou, China

**Keywords:** ovarian carcinoma, ferroptosis, mRNA, therapeutic targets, prognosis, tumor microenvironment

## Abstract

**Objective:** This study aimed to explore ferroptosis-related mRNAs as potential therapeutic targets for ovarian cancer treatment.

**Methods:** Molecular subtypes were classified based on ferroptosis-related mRNAs via ConsensusClusterPlus package. The differences in prognosis, stromal score, immune score, immune function, and immune checkpoints were assessed between subtypes. Small molecular drugs were predicted via the CMap database. The sensitivity to chemotherapy drugs was estimated through the GDSC. A LASSO Cox regression model was conducted via the glmnet package, followed by a nomogram model.

**Results:** Based on ferroptosis mRNA expression profile, two molecular subtypes (C1 and C2) were classified, with distinct clinical outcomes. C1 subtype exhibited higher stromal score, immune cell score (T helper, Treg, neutrophil) and immune function (APC co-inhibition, parainflammation and Type II IFN response). Higher mRNA expression levels of immune checkpoints (like PDCD1) were found in C1 than C2. Potential small molecular drugs (PI3K and mTOR inhibitors) were found for treatment of ovarian cancer. C1 was more sensitive to eight chemotherapy drugs (A.443654, AZD.0530, AZD6482, AZD7762, AZD8055, BAY.61.3606, Bicalutamide, and CGP.60474). A 15-ferroptosis-related mRNA signature was developed, which could robustly and independently predict the outcomes. Moreover, a nomogram was established combining the signature and age, which could intuitively and accurately predict the 5-year overall survival probability.

**Conclusion:** Our study characterized two ferroptosis-related subtypes with distinct prognosis and tumor immune features, which could assist clinicians make decisions and individual therapy. Moreover, 15 ferroptosis-related mRNAs were identified, which could become potential therapeutic targets for ovarian cancer.

## Introduction

Ovarian carcinoma is a highly lethal gynecological malignancy globally (1). Because early symptoms are rare, ovarian cancer is usually diagnosed as advanced, partly leading to unfavorable clinical outcomes, with a 5-year overall survival (OS) of 45% (2). So far, ovarian cancer is mainly classified according to histology, grade, and stage. Nevertheless, molecular features of genome, transcription, translation, and post-translational modifications contribute to the heterogeneity of ovarian cancer (3). Characterization of specific molecular subtypes may assist clinicians make decisions and individual therapy.

It is of importance to discover and intervene risk factors via effective cancer prevention during cancer progression (4). Currently, various driver genes have been found. Recent findings demonstrate that ferroptosis, a novel cell death type with an iron-dependent mechanism, may participate in cancer progression (5). Activation of ferroptosis remarkably suppresses the proliferative capacity of ovarian cancer cells (6), indicating that inducing ferroptosis is a promising therapeutic strategy for ovarian cancer. The mechanism of ferroptosis is still largely unknown. Recent research has found that ferroptosis can be activated by TAZ-ANGPTL4-NOX2 axis for ovarian cancer, which provides a ferroptosis-inducing therapeutic implication (2). Immunotherapy such as immune checkpoint inhibitors has been applied in the treatment of ovarian cancer (7). It is clinical importance to probe which factors affect the outcomes of immunotherapy. Increasing evidence highlights the critical roles of ferroptosis on immune evasion (8). In turn, CD8 + T cells induce tumor ferroptosis in immunotherapy (9). Targeting ferroptosis combined with immunotherapy may be an underlying therapeutic strategy. Nevertheless, the roles and prognostic values of ferroptosis mRNAs remain to be illustrated in ovarian cancer.

Accordingly, this study established tumor subtypes for ovarian cancer on the basis of ferroptosis mRNAs. Furthermore, 15 ferroptosis-related mRNAs were identified as potential therapeutic targets for ovarian cancer.

## Materials and Methods

### Patients and Specimens

The mRNA expression data and matched clinical information were retrieved from The Cancer Genome Atlas (TCGA; https://portal.gdc.cancer.gov/) database on November 23, 2020. Following removing samples with incomplete follow-up information, 379 samples were retained, as the training cohort. A microarray ovarian carcinoma dataset (accession: GSE26193) was obtained from the Gene Expression Omnibus (GEO; https://www.ncbi.nlm.nih.gov/geo/) database on the GPL570 platform. If a gene ID corresponded to multiple probes, the expression value was averaged. The GSE26193 dataset was utilized as the validation cohort.

### Consensus Clustering

Consensus clustering, an unsupervised clustering method, classifies samples based on the mRNA expression profiles. Herein, ovarian carcinoma samples were clustered on the basis of the expression levels of ferroptosis-related mRNAs (Supplementary Table 1) using the ConsensusClusterPlus package in R (version 1.54.0) (10). Re-sampling was used to sample 80% of the samples. After multiple sampling, the optimal *k* value was identified when the number of clusters *k* = 2, 3, 4, ·····9. The optimal k value was determined when the cumulative distribution function (CDF) index was up to the approximate maximum. The classification was verified by principal components analysis (PCA) based on the mRNA expression profiles of ovarian cancer.

### Estimation of Stromal and Immune Cells in Malignant Tumors Using Expression Data (ESTIMATE)

Tumor microenvironment is composed of tumor cells, stromal cells, immune cells, and the like. The ESTIMATE (version 2.0.0) package in R was applied for evaluation of the stromal score, immune score, and tumor purity in each ovarian cancer tissue sample.

### Connectivity Map (CMap)

Differentially expressed genes (DEGs) were filtered between two tumor subtypes through the limma package with the threshold values of false discovery rate < 0.05 and |fold change| ≥1.5. The two lists of up- and down-regulated genes were input into the CMap (http://portals.broadinstitute.org/cmap/) database (11). Potential small molecule drugs were predicted based on the enrichment values as well as permutation *p*-values. Moreover, underlying mechanisms of action were probed via the CMap mode-of-action (MoA) analysis.

### Single Sample Gene Set Enrichment Analysis (ssGSEA)

The ssGSEA method was utilized to quantify the enrichment scores of immune cells (aDCs, B cells, CD8+ T cells, DCs, iDCs, macrophages, mast cells, neutrophils, NK cells, pDCs, T helper cells, Tfh, Th1 cells, Th2 cells, TIL and Treg), and immune functions (APC co-inhibition, APC co-stimulation, CCR, check-point cytolytic activity, HLA, inflammation-promoting, MHC class I, parainflammation, T cell co-inhibition, T cell co-stimulation, type I IFN response and type II IFN response) for each ovarian cancer specimen (12).

### Drug Sensitivity Prediction

The sensitivity to chemotherapy drugs was estimated through the Genomics of Drug Sensitivity in Cancer (GDSC; https://www.cancerrxgene.org/) database (13). The half maximal inhibitory concentration (IC50) was estimated using the pRRophetic package in R (14).

### Construction of a Ferroptosis-Related Signature

Univariate Cox regression analysis was applied to screen prognosis-related ferroptosis mRNAs with *p* < 0.05 through the survival package in R (version 2.41–3). The key mRNAs that affected clinical outcomes of patients were then screened by the least absolute shrinkage and selection operation (LASSO) Cox regression model, which was achieved by the glmnet package (version 3.0–1) (15). The regression coefficient was calculated for each variable via the multivariate Cox regression analysis. The risk score was determined for each ovarian cancer patient by combining the regression coefficients and expression levels of the key mRNAs. The cutoff point was determined according to the median value of risk scores. Afterwards, patients in the training and validation cohorts were separated into high- and low-risk groups. The survival differences were evaluated between the two groups.

### Nomogram

A nomogram model was established by combining variables that could independently predict the survival of ovarian cancer. The predictive efficacy of the model for prediction of 5-year overall survival probability was assessed through calibration plots via the rms package in R.

### Gene Expression Profiling Interactive Analysis (GEPIA)

The expression levels of 15 ferroptosis-related mRNAs in the signature were analyzed in ovarian cancer (*n* = 426) and normal specimens (*n* = 88) from match TCGA normal and GTEx data via the GEPIA website (http://gepia2.cancer-pku.cn/#index).

### Immunohistochemistry

Immunohistochemistry images of 15 ferroptosis-related proteins in ovarian cancer tissues from the signature model were downloaded from the Human Protein Atlas (https://www.proteinatlas.org/).

### RT-qPCR

Twenty pairs of ovarian cancer and adjacent normal tissues were collected from the Department of Obstetrics and Gynecology of Cangzhou Central Hospital (China). None of the patients received any treatment before surgery. Each patient signed a written informed consent. This study was approved by the Ethics Committee of Cangzhou Central Hospital (2019045). Total RNA from tissues was extracted by TRIzol reagent (Beyotime, Beijing, China), which was reverse-transcribed into cDNA. RT-PCR for mRNAs was carried out through SYBR Premix Ex Taq reagent kit (Invitrogen, USA) on the ABI StepOne RT-PCR System. The mRNA expression was normalized against GAPDH. Primer sequences were listed in Table 1.

**Table 1:** Primer sequences for RT-qPCR.

**Target genes**	**Primer sequences (5′-3′)**
CDKN1B	AACGTGCGAGTGTCTAACGG (forward) CCCTCTAGGGGTTTGTGATTCT (reverse)
FAS	TCTGGTTCTTACGTCTGTTGC (forward) CTGTGCAGTCCCTAGCTTTCC (reverse)
FOS	CCGGGGATAGCCTCTCTTACT (forward) CCAGGTCCGTGCAGAAGTC (reverse)
FOXO1	TCGTCATAATCTGTCCCTACACA (forward) CGGCTTCGGCTCTTAGCAAA (reverse)
GABARAPL1	ATGAAGTTCCAGTACAAGGAGGA (forward) GCTTTTGGAGCCTTCTCTACAAT (reverse)
HDAC1	CTACTACGACGGGGATGTTGG (forward) GAGTCATGCGGATTCGGTGAG (reverse)
NFKB1	AACAGAGAGGATTTCGTTTCCG (forward) TTTGACCTGAGGGTAAGACTTCT (reverse)
PEX3	CCAAGCACGACGACAATATCA (forward) TCAGTGTTGGAAGCATGGACA (reverse)
PPP1R15A	ATGATGGCATGTATGGTGAGC (forward) AACCTTGCAGTGTCCTTATCAG (reverse)
SIRT2	TGCGGAACTTATTCTCCCAGA (forward) GAGAGCGAAAGTCGGGGAT (reverse)
CXCR4	ACTACACCGAGGAAATGGGCT (forward) CCCACAATGCCAGTTAAGAAGA (reverse)
IFNG	TCGGTAACTGACTTGAATGTCCA (forward) TCGCTTCCCTGTTTTAGCTGC (reverse)
IL24	TTGCCTGGGTTTTACCCTGC (forward) AAGGCTTCCCACAGTTTCTGG (reverse)
MTMR14	GGAGTTCTCCCGGACTCAGTA (forward) AACAGTAGTCTCGGCCAAACA (reverse)
RB1	CTCTCGTCAGGCTTGAGTTTG (forward) GACATCTCATCTAGGTCAACTGC (reverse)
GAPDH	ACAACTTTGGTATCGTGGAAGG (forward) GCCATCACGCCACAGTTTC (reverse)

### Statistical Analysis

Statistical analyses were conducted through R language (version 3.6.2) or SPSS (version 19.0). Differences between two groups were analyzed via the Wilcoxon test or student's *t*-test. Kaplan-Meier curves were utilized for measurement of survival rates for OS and disease-free survival (DFS), and differences in survival rates were presented via the log-rank test. The predictive efficacy of signature was estimated by time-dependent receiver operating characteristic curves (ROCs). The area under the curves (AUCs) was calculated. Univariate and multivariate cox regression analyses were employed to probe whether clinical features (age, FIGO stage, and grade) and signature were associated with prognosis of ovarian cancer. Hazard ratio (HR) and 95% confidence interval (CI) of each variable were estimated using the survminer package.

## Results

### Two Molecular Subtypes of Ferroptosis-Related mRNAs in Ovarian Cancer

**Figure 1:**
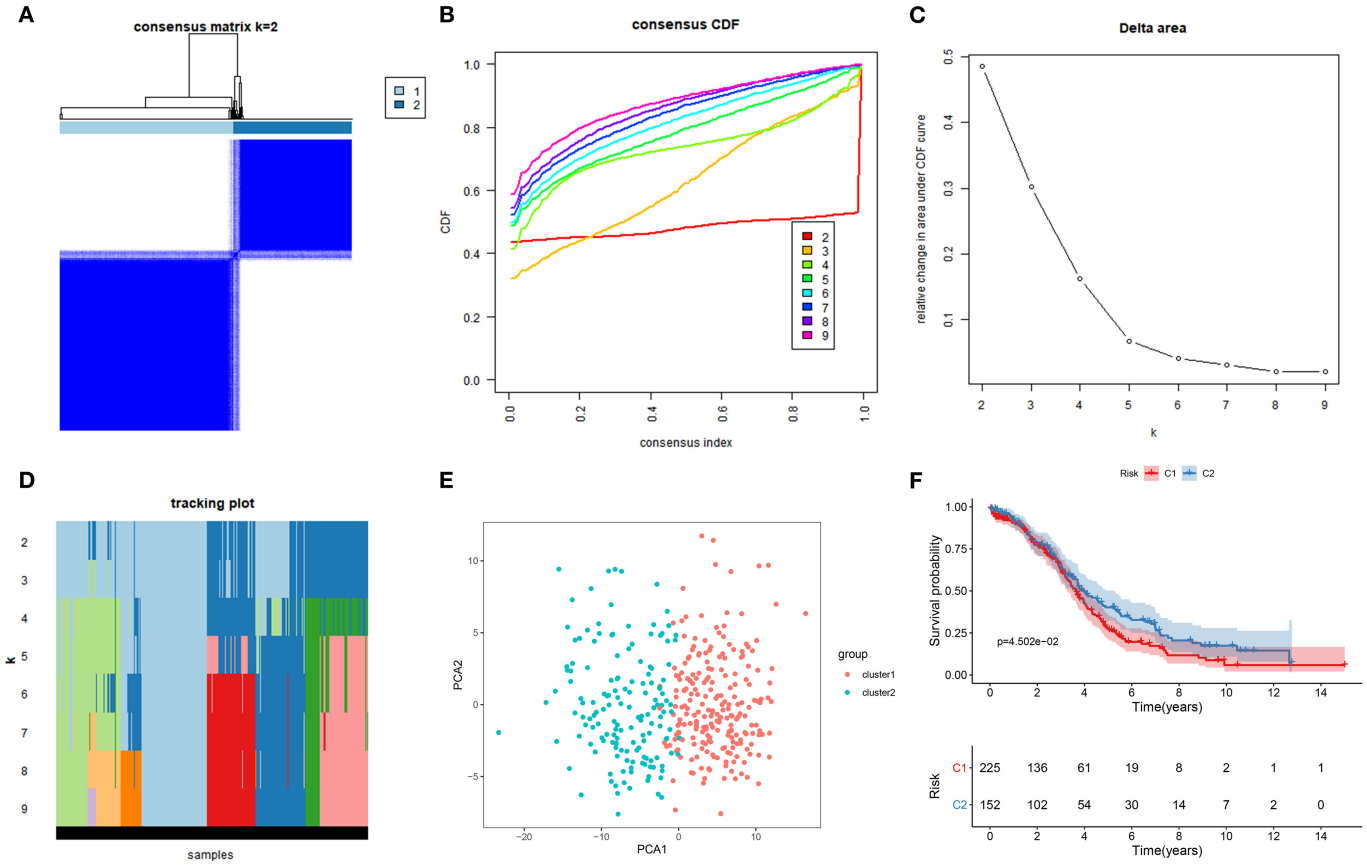
Characterization of two molecular subtypes based on ferroptosis-related mRNAs in ovarian cancer. **(A)** Consensus matrix when *k* = 2. Both the rows and columns of the matrix represent samples. From white to dark blue, the value of the consistency matrix is from 0 to 1. **(B)** Consensus CDF, **(C)** Delta area, and **(D)** tracking plot for validation of the clustering results. **(E)** PCA plots for validation of the stability and reliability of the classification. **(F)** Kaplan-Meier OS curves for ovarian cancer patients between C1 and C2 subtypes.

This study classified molecular subtypes based on the expression profiles of ferroptosis-related mRNAs in ovarian cancer via the ConsensusClusterPlus package in the TCGA ovarian cancer dataset. When *k* = 2, the classification was reliable and stable (Figures 1A–D). The patients were divided into C1 and C2. The PCA confirmed that C1 was significantly different from C2 (Figure 1E). Patients in C2 exhibited the prolonged survival time than those in C1 (*p* = 4.502e-02; Figure 1E). Thus, ferroptosis-based signatures characterized two tumor subtypes with distinct clinical outcomes for ovarian cancer.

### Association Between Two Subtypes and Tumor Immune Microenvironment

**Figure 2:**
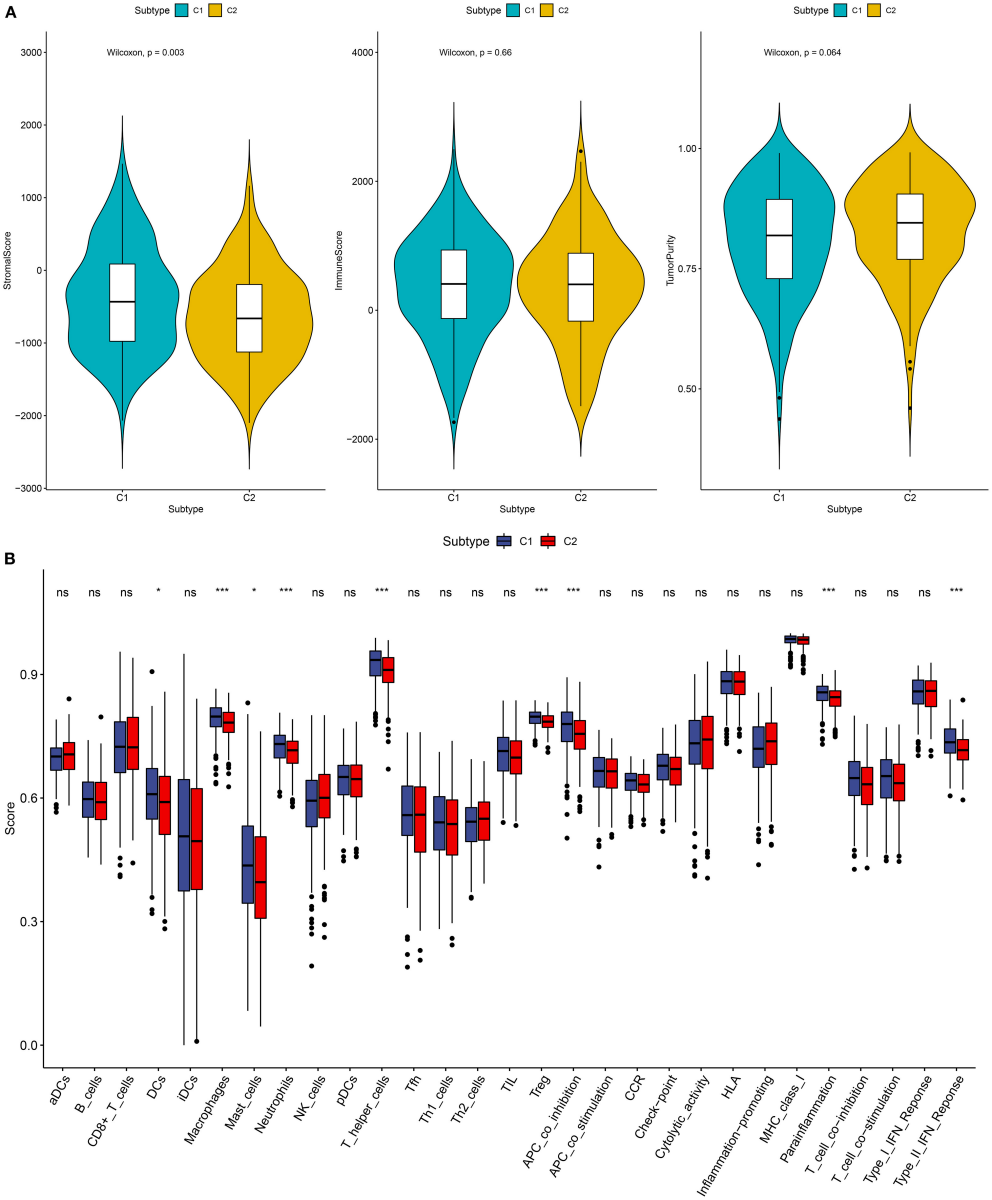
Association between two subtypes and tumor immune microenvironment. **(A)** Violin diagrams for depicting the associations between subtypes and stromal score, immune score, and tumor purity in ovarian cancer. **(B)** Box plots showing the relationships between subtypes and immune cell infiltrations and functions. Ns, not significant; *p < 0.05; ***p < 0.001.

Recently, it has been demonstrated the crosstalk between ferroptosis and tumor immune microenvironment in cancers (16). Therefore, we analyzed the associations between two subtypes and tumor immune microenvironment. The stromal score, immune score and tumor purity of each ovarian cancer sample were calculated via the ESTIMATE. We found that there was a significant correlation in stromal score between C1 and C2 (*p* = 0.003; Figure 2A). C2 had a distinctly higher stromal score than C1. However, no significant differences in immune score and tumor purity were found between two subtypes. Furthermore, we assessed whether subtypes were in relation to immune cells and immune functions in ovarian cancer. The data showed that C1 exhibited significantly higher infiltration levels of DCs, macrophages, mast cells, neutrophils, T helper cells, and Treg than C2 (Figure 2B). Moreover, the relationships between subtypes and immune functions were assessed in depth. APC co-inhibition, parainflammation, and type II IFN response exhibited higher levels in C1 compared to C2. Taken together, two subtypes had a relationship with tumor immune microenvironment in ovarian cancer.

### Association Between Subtypes and Immune Checkpoints in Ovarian Cancer

**Figure 3:**
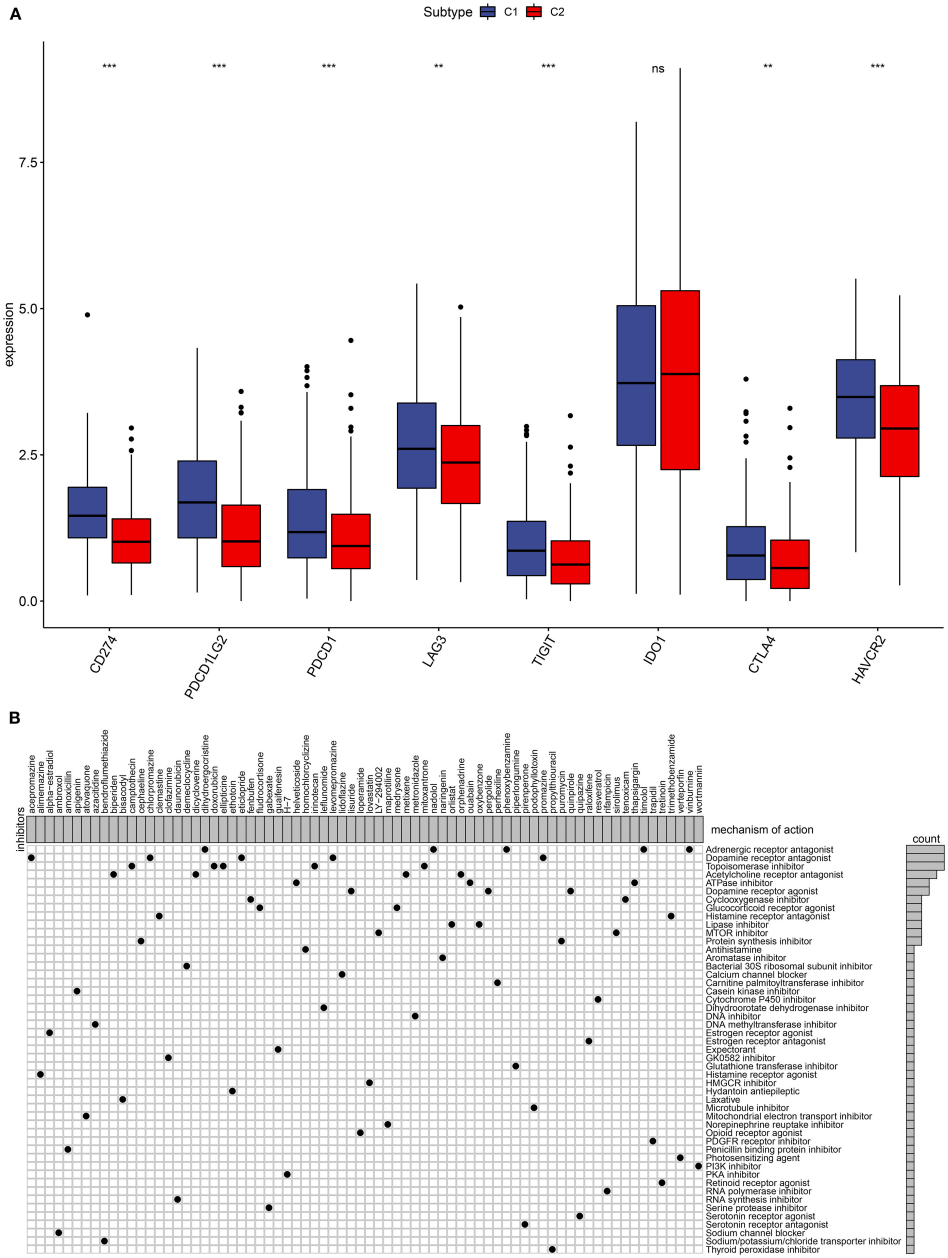
Immune checkpoints and small molecular drugs in ovarian cancer. **(A)** Box plots for the associations between subtypes and immune checkpoints in ovarian cancer. Ns, not significant; **p < 0.01; ***p < 0.001. **(B)** Heatmap showing small inhibitors (perturbagen) and their shared mechanisms of action (rows) from the CMap database.

The immune checkpoint inhibitors have been used in ovarian cancer (7). Herein, we found that C1 had significantly higher CD274, PDCD1LG2, PDCD1, LAG3, TIGIT, CTLA4, and HAVCR2 compared to C2, indicating that patients in C1 could be sensitive to the immune checkpoint inhibitors (Figure 3A).

### Analysis of Small Drugs and Mechanism of Action Between Subtypes

Four thousand two hundred seventy-five up- and 264 down-regulated genes were screened between two subtypes. Based on them, CMap analysis was utilized to identify candidate small molecular drugs for ovarian cancer. For example, mTOR (LY-294002 and sirolimus) and PI3K inhibitors (wortmannin) were predicted for the treatment of ovarian cancer (Figure 3B).

### Sensitivity of Chemotherapy Drugs Between Subtypes

**Figure 4:**
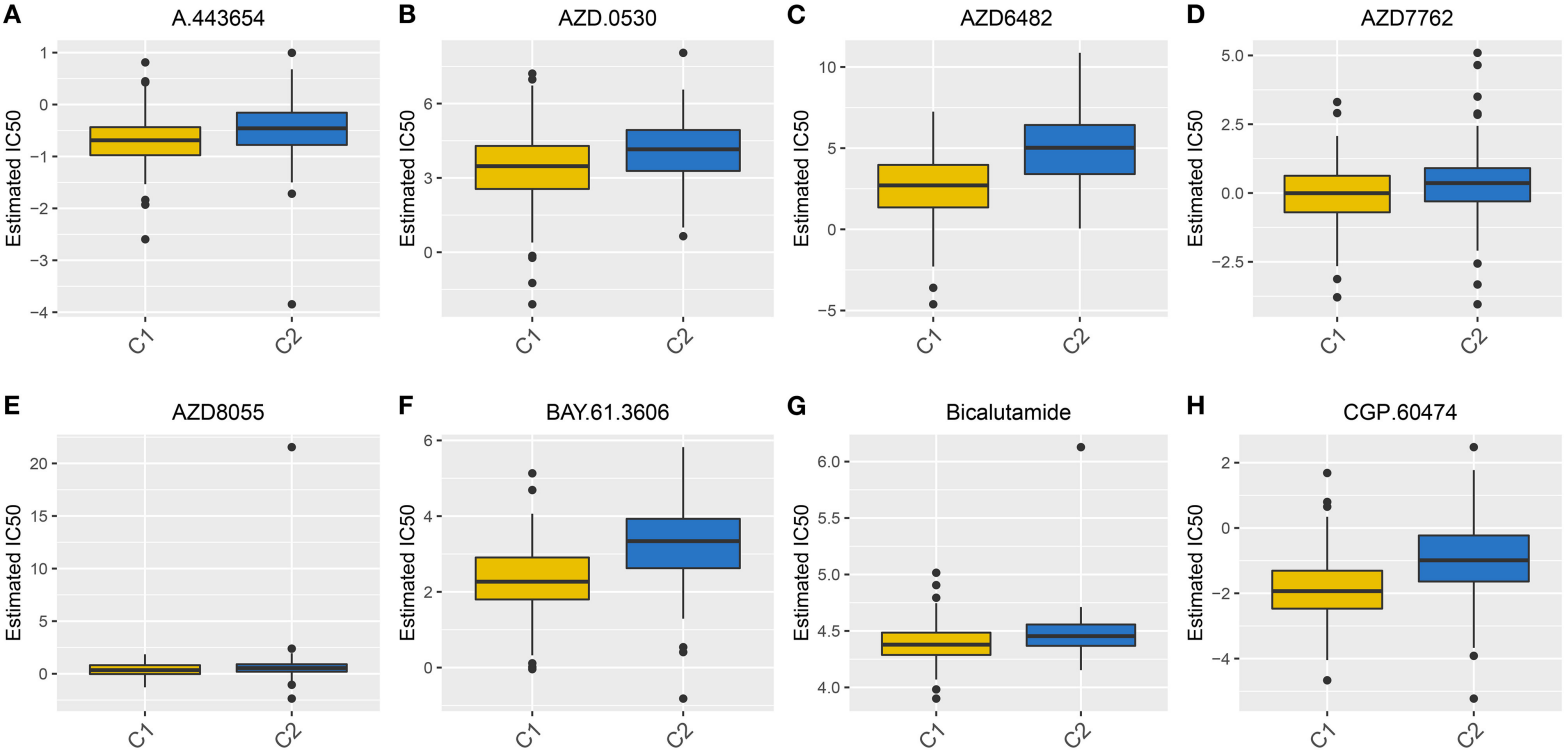
Box plots for the estimated IC50 of chemotherapy drugs between two subtypes. **(A)** A.443654. **(B)** AZD.0530. **(C)** AZD6482. **(D)** AZD7762. **(E)** AZD8055. **(F)** BAY.61.3606. **(G)** Bicalutamide. **(H)** CGP.60474.

Radical surgery in combination with adjuvant chemotherapy is the basic approach regarding ovarian cancer. Thus, it is of significance for predicting the sensitivity to chemotherapy, which may assist clinicians to employ the optimal strategy. The estimated IC50 levels of A.443654 (*p* = 2.67e-07), AZD.0530 (*p* = 1.08e-07), AZD6482 (*p* = 2.16e-24), AZD7762 (*p* = 0.001), AZD8055 (*p* = 0.02), BAY.61.3606 (*p* = 7.54e-19), Bicalutamide (*p* = 3.05e-08), and CGP.60474 (*p* = 6.82e-17) were distinctly lowered in C1 compared to C2 (Figures 4A–H), indicating that C1 subtype was more sensitive to these chemotherapeutic drugs.

### Establishment of a Ferroptosis-Related Signature for Prognosis Prediction

**Figure 5:**
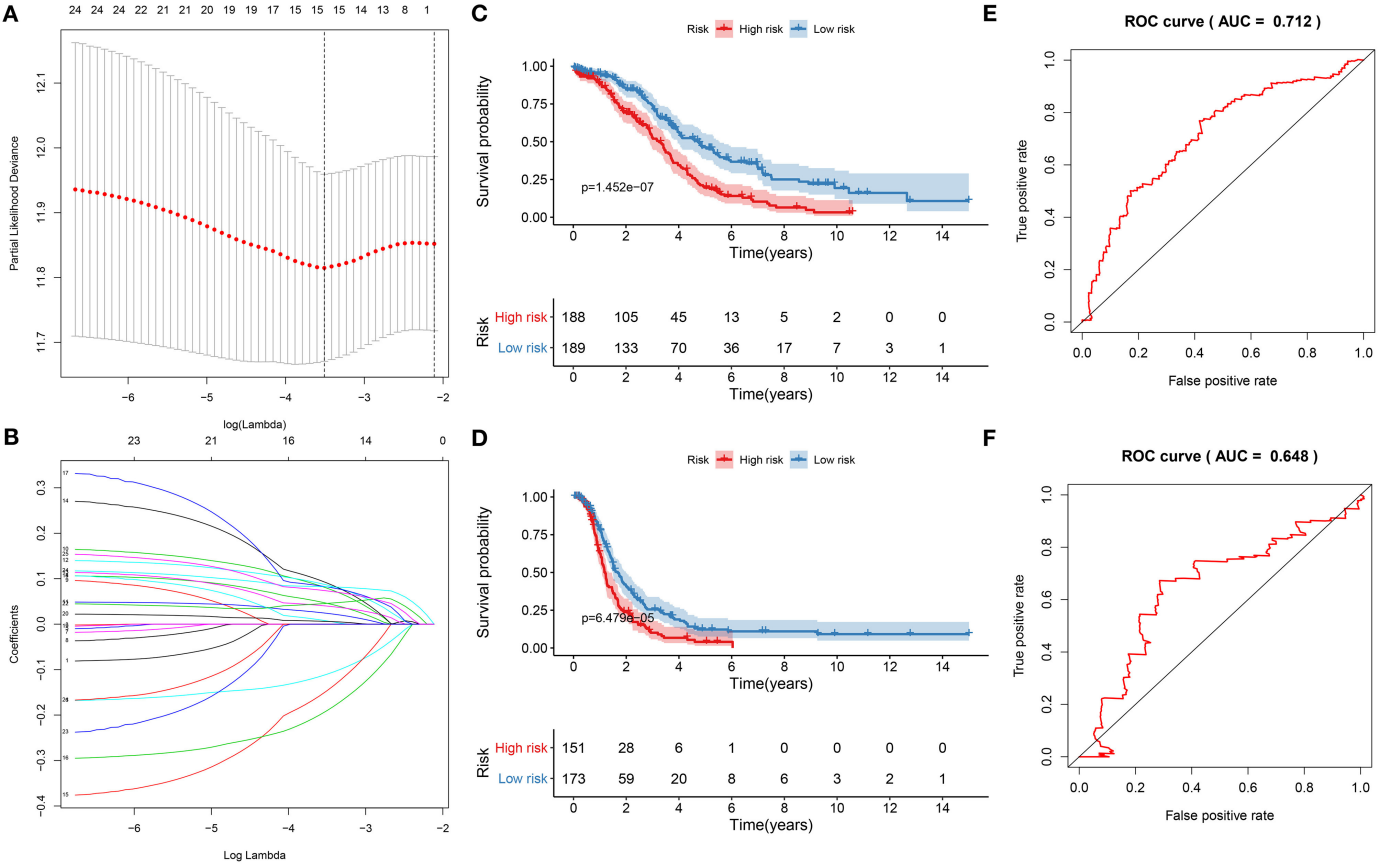
Construction of a ferroptosis-related signature for prediction of prognosis in ovarian cancer. **(A)** LASSO regression with 10-fold cross-verification. **(B)** LASSO coefficient profiles of 25 ferroptosis-related mRNAs. Kaplan-Meier curves for **(C)** OS and **(D)** DFS between high and low risk groups. Time-dependent ROC curves for **(E)** OS and **(F)** DFS.

Twenty-five ferroptosis mRNAs were distinctly related to prognosis of ovarian cancer (Supplementary Table 2). After screening the key mRNAs, a LASSO Cox regression model was then constructed (Figures 5A,B). The risk score for each patient was calculated, as follows: 0.0461419186912939 * CDKN1B +−0.110510609535903 * CXCR4 + 0.0795555188969791 * FAS + 0.0239651512531641 * FOS + 0.0864413738704437 * FOXO1 + 0.0399109420909256 * GABARAPL1 + 0.0910556896121155 * HDAC1 +−0.147263024908922 * IFNG + (−0.190901967548489) * IL24 + 0.0821435843345801 * MTMR14 + 0.00734788035781478 * NFKB1 + 0.00606909390683674 * PEX3 + 0.0456165637799593 * PPP1R15A + 0.0822296594101889 * RB1 + 0.0733586200221587 * SIRT2. Ovarian cancer patients were separated into high and low risk groups. Our results showed that patients with high risk exhibited shorter OS (*p* = 1.452e-07; Figure 5C) and DFS time (*p* = 6.479e-05; Figure 5D) than those with low risk. The predictive efficacy of this signature was verified by ROCs. The AUCs for OS (Figure 5E) and DFS (Figure 5F) were 0.712 and 0.648, suggesting that the signature possessed the accurate and robust performance for prediction of prognosis.

### Evaluation of the Stability and Reliability of the Signature for Prediction of Prognosis in Ovarian Cancer

**Figure 6:**
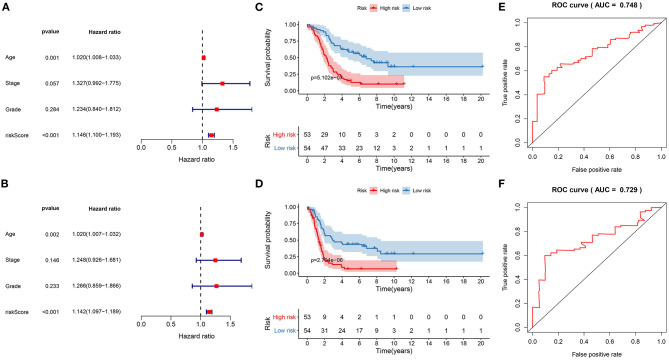
Evaluation of the stability and reliability of the signature for prediction of prognosis in ovarian cancer. **(A)** Univariant and **(B)** multivariate cox regression analysis of age, stage, grade, and risk score in the training set. Kaplan-Meier curves for **(C)** OS and **(D)** DFS of risk score in the GSE26193 dataset. ROC curves for **(E)** OS and **(F)** DFS of risk score in the GSE26193 dataset.

Univariate cox regression analysis results showed that the risk score was significantly associated with poor prognosis (*p* = 5.279e-11, HR: 1.146, CI: 1.100–1.193) for ovarian cancer patients (Figure 6A). Furthermore, age was a risk score for ovarian cancer prognosis (*p* = 0.001, HR: 1.020, CI: 1.008–1.033). To verify the efficacy of these factors on prediction of prognosis, multivariate cox regression analysis was carried out. Our data suggested that the risk score (*p* = 1.460e-10, HR: 1.142, CI: 1.097–1.189) and age (*p* = 0.002, HR: 1.020, CI: 1. 007–1.032) were independent risk factors for ovarian cancer (Figure 6B). To further verify the generalizability of this signature, the predictive performance was externally validated in the GSE26193 dataset. Consistent with the training set, the signature was markedly associated with OS (*p* = 5.102e-07; Figure 6C) and DFS (*p* = 2.704e-06; Figure 6D). ROCs confirmed the stability and reliability of the signature for prediction of OS (AUC = 0.748; Figure 6E) and DFS (AUC = 0.729; Figure 6F). Hence, this signature could be a stable and reliable risk factor for ovarian cancer.

### Construction of a Nomogram Model Based on the Risk Score and Age

**Figure 7:**
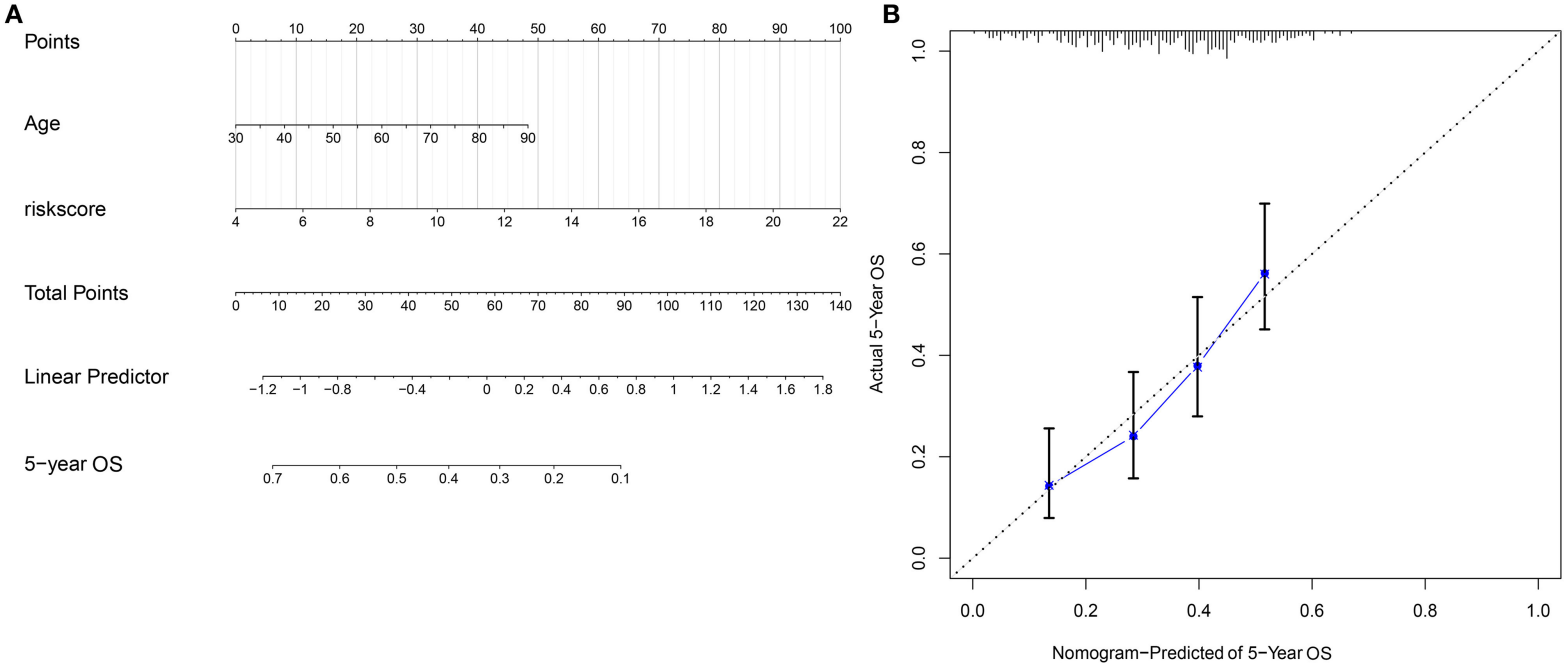
Construction and evaluation of a nomogram combining the risk score and age. **(A)** A nomogram for prediction of 5-year OS probability. **(B)** Calibration plots for assessing the relationships between predicted 5-year overall survival and actual survival time.

Through combining the two independent risk factors (signature and age), we established a nomogram model for predicting the clinical outcomes of ovarian cancer. The risk score made the most contribution to the prediction of 5-year OS time (Figure 7A). The prediction efficacy of the nomogram was assessed by calibration plots. The data showed that the 5-year OS predicted by the nomogram was close to the actual survival time (Figure 7B). It was indicative of the superior predictive capacity of the nomogram.

### 15 Ferroptosis-Related mRNAs as Potential Therapeutic Targets for Ovarian Cancer

**Figure 8:**
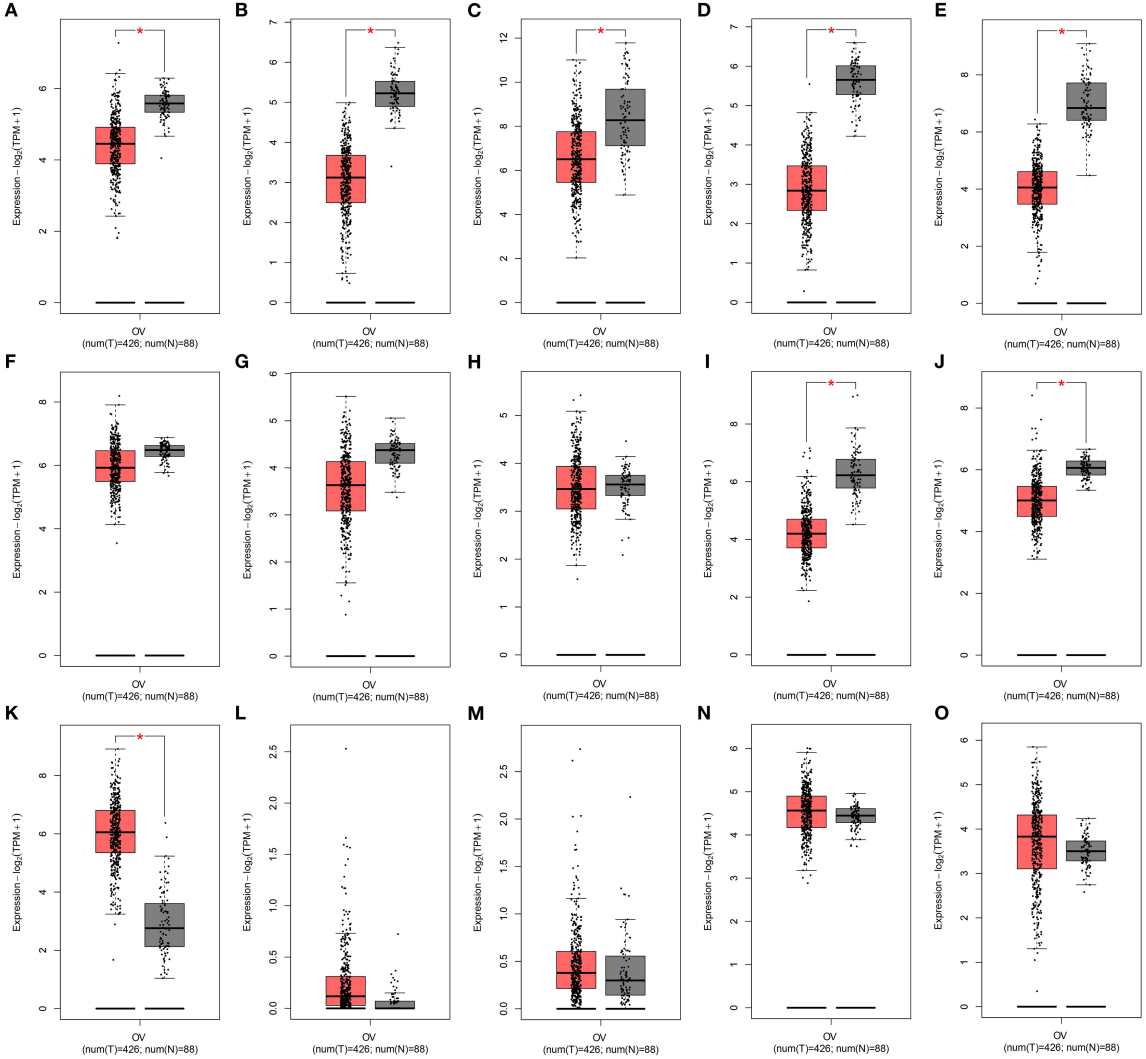
Expression of the 15 ferroptosis-related mRNAs from the signature in an ovarian cancer cohort from the GEPIA database. Box plots for the expression of **(A)** CDKN1B; **(B)** FAS; **(C)** FOS; **(D)** FOXO1; **(E)** GABARAPL1; **(F)** HDAC1; **(G)** NFKB1; **(H)** PEX3; **(I)** PPP1R15A; **(J)** SIRT2; **(K)** CXCR4; **(L)** IFNG; **(M)** IL24; **(N)** MTMR14; **(O)** RB1 in ovarian cancer and normal tissues.

The expression of the 15 mRNAs from the signature was analyzed in TCGA ovarian cancer cohort. We found that CDKN1B, FAS, FOS, FOXO1, GABARAPL1, HDAC1, NFKB1, PEX3, PPP1R15A, and SIRT2 were all significantly lowly expressed in ovarian cancer than normal specimens (Figures 8A–J). Moreover, CXCR4, IFNG, IL24, MTMR14, and RB1 all exhibited higher expression in ovarian cancer compared to normal specimens (Figures 8K–O). These data indicated that these ferroptosis-related mRNAs were associated with ovarian cancer progression.

### Expression of Gene Products From the 15-mRNA Model in Ovarian Cancer

**Figure 9:**
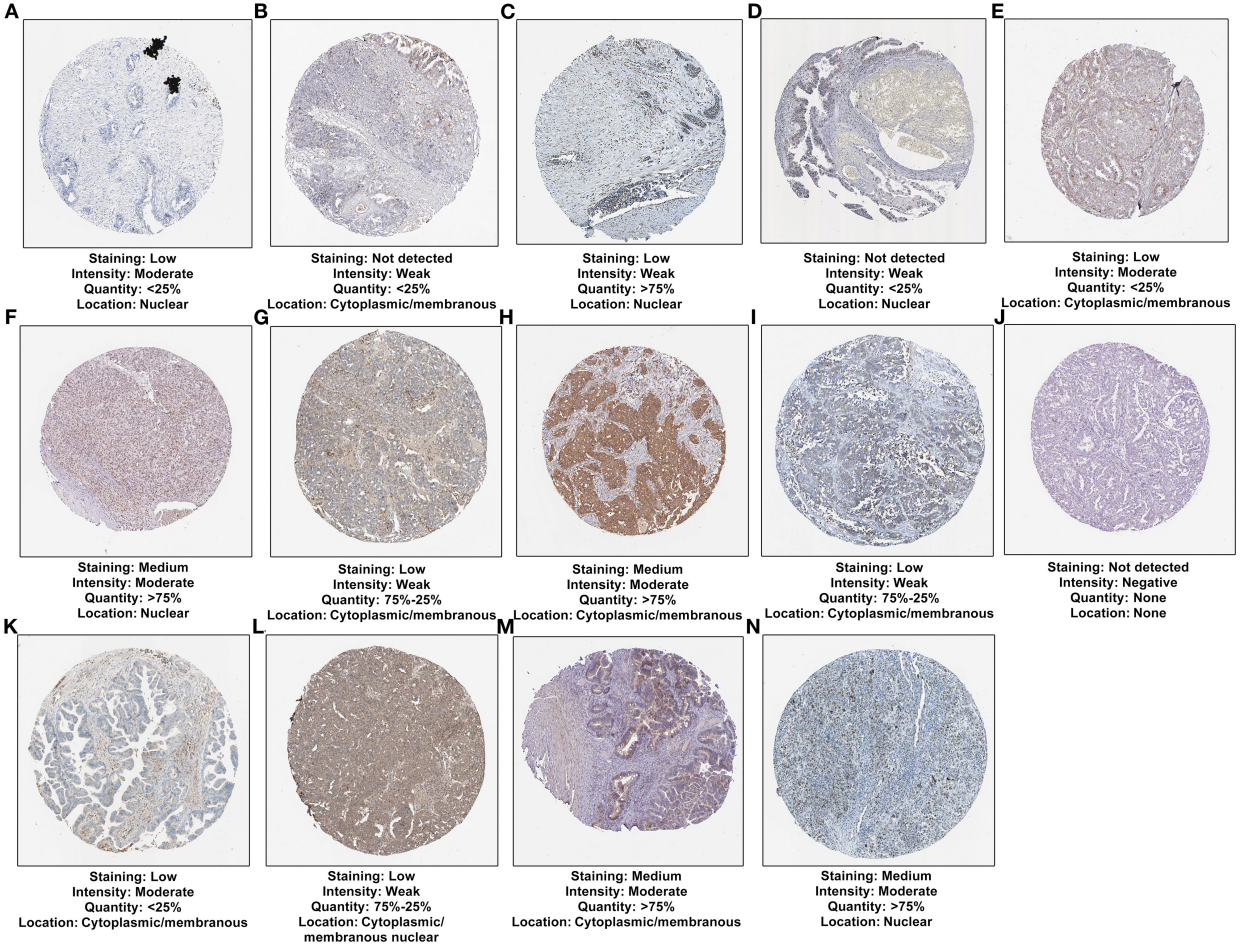
Verification of the expression of 14 mRNAs from the risk score at the translation levels. **(A)** CDKN1B; **(B)** FAS; **(C)** FOS; **(D)** FOXO1; **(E)** GABARAPL1; **(F)** HDAC1; **(G)** NFKB1; **(H)** PEX3; **(I)** PPP1R15A; **(J)** SIRT2; **(K)** IFNG; **(L)** IL24; **(M)** MTMR14; **(N)** RB1.

We analyzed the expression of gene products in the 15-mRNA signature in ovarian cancer tissues. Among them, CXCR4 was not contained in the database. The immunohistochemistry images of CDKN1B, FAS, FOS, FOXO1, GABARAPL1, HDAC1, NFKB1, PEX3, PPP1R15A, SIRT2, IFNG, IL24, MTMR14, and RB1 in ovarian cancer tissues were shown in Figures 9A–N. This study found that the expression intensity of CDKN1B, GABARAPL1 and IFNG was moderate and the quantity was low. The expression intensity and quantity of FAS and FOXO1 were both low. For FOS, NFKB1, PPP1R15A, and IL24, the intensity was weak, but the quantity was low to high. HDAC1, PEX3, MTMR14, and RB1 had the moderate intensity and high quantity in ovarian cancer. SIRT2 expression was not detected in ovarian cancer. CDKN1B, FOS, FOXO1, HDAC1, and RB1 were mainly expressed in the nuclear of tumor cells. Meanwhile, FAS, GABARAPL1, NFKB1, PEX3, PPP1R15A, IFNG, and MTMR14 were primarily distributed in cytoplasmic and membranous regions. IL24 was mainly distributed in cytoplasmic and membranous nuclear.

### Validation of 15 Ferroptosis-Related mRNAs in Ovarian Cancer by RT-qPCR

**Figure 10:**
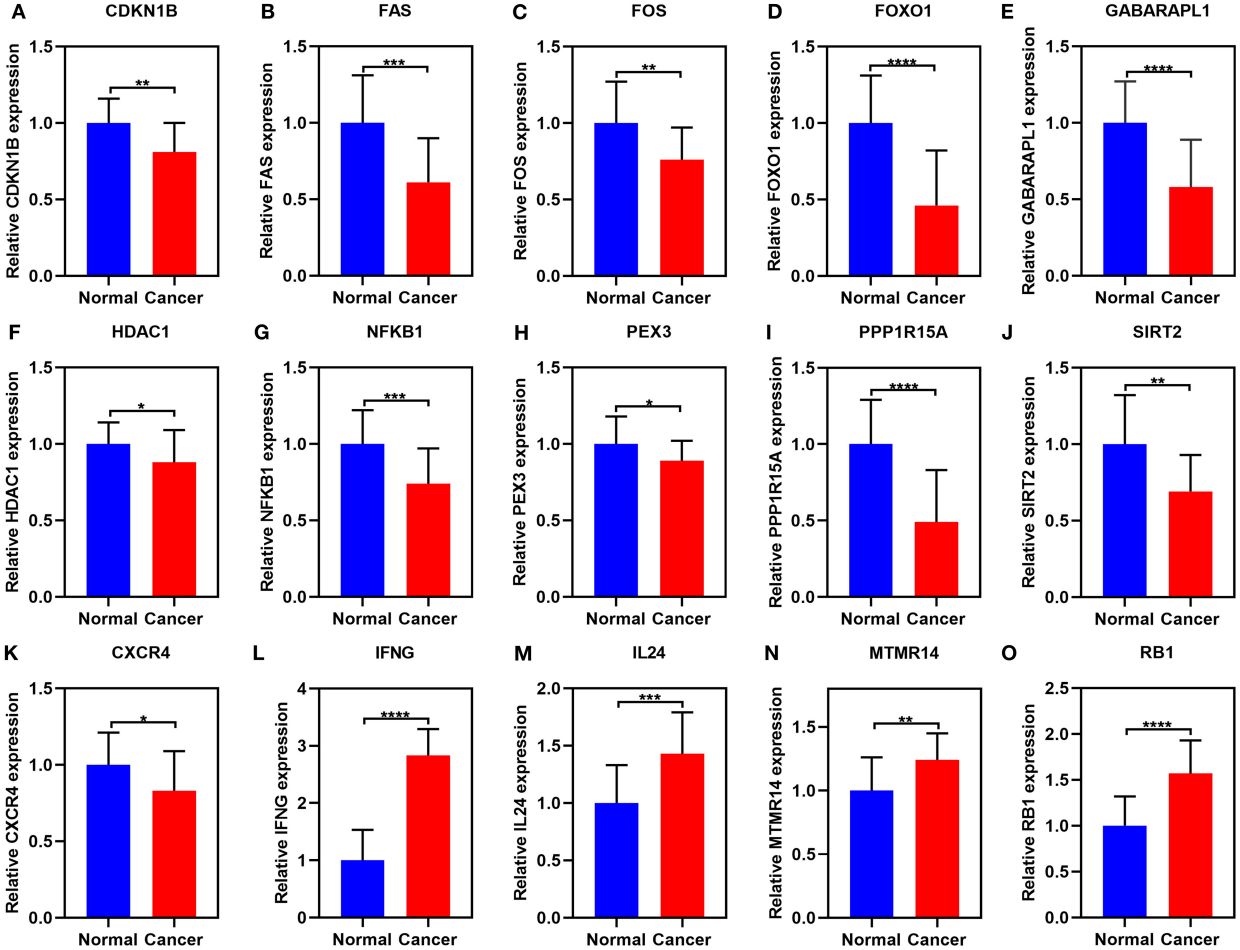
RT-qPCR for the expression of the 15 ferroptosis-related mRNAs in ovarian cancer and normal tissues. **(A)** CDKN1B; **(B)** FAS; **(C)** FOS; **(D)** FOXO1; **(E)** GABARAPL1; **(F)** HDAC1; **(G)** NFKB1; **(H)** PEX3; **(I)** PPP1R15A; **(J)** SIRT2; **(K)** CXCR4; **(L)** IFNG; **(M)** IL24; **(N)** MTMR14; **(O)** RB1. *p < 0.05; **p < 0.01; ***p < 0.001; ****p < 0.0001.

These 15 ferroptosis-related mRNAs were verified in 20 paired ovarian cancer and normal tissues by RT-qPCR. Our data confirmed the down-regulation of CDKN1B, FAS, FOS, FOXO1, GABARAPL1, HDAC1, NFKB1, PEX3, PPP1R15A, SIRT2, and CXCR4 in ovarian cancer than normal tissues (Figures 10A–K). Furthermore, IFNG, IL24, MTMR14, and RB1 were highly expressed in ovarian cancer than normal tissues (Figures 10L–O).

## Discussion

Ovarian cancer, with high heterogeneity, has various subtypes based on histological and molecular features (17). Herein, two tumor subtypes with distinct prognosis were characterized by ferroptosis-related signatures for ovarian cancer, which highlighted the diversity among ovarian cancer samples.

In this study, two molecular subtypes (C1 and C2) of ovarian cancer were defined through the mRNA expression profiles of ferroptosis-related mRNAs. Ovarian cancer samples are composed of cancer and stromal cells (18). There exists the tight crosstalk between cancer and stromal cells in tumor microenvironment (19). Targeting this crosstalk is a hopeful therapeutic approach (20). Our data demonstrated that C2 had a distinctly higher stromal score than C1. It has been found that cancer-associated stroma contributes to poor outcomes for high-grade serous ovarian cancer (18). T cell dysfunction is a hallmark of cancers (21). Nevertheless, the mechanisms of dysfunction remain unclear. We found that C1 exhibited significantly higher infiltration levels of T helper and Treg cells than C2, indicating that ferroptosis might be related to T cell dysfunction. Previously, T cells can activate tumor ferroptosis in immunotherapies (9). Moreover, C1 had higher infiltration levels of DCs, macrophages, mast cells, neutrophils compared to C2. Polarization of tumor-associated macrophages is driven by autophagy-dependent ferroptosis (22). Furthermore, ferroptosis can be induced neutrophils, thereby promoting tumor necrosis in glioblastoma (23). Our results suggested that APC co-inhibition, parainflammation, and type II IFN response had higher levels in C1 compared to C2. Ferroptosis can be induced by interferon-γ in cancer cells (24). The immune checkpoints including CD274, PDCD1LG2, PDCD1, LAG3, TIGIT, CTLA4, and HAVCR2 exhibited higher levels in C1 compared to C2. Immunity therapy such as anti-PD-1 and ani-PD-L1 is representative of the tomorrow of cancer treatment. Consistently, induction of ferroptosis tumor cells at early stage may efficiently boost immunity response (25).

Exploitation of ferroptosis inducers offers a novel therapeutic approach for treating ovarian cancer. A few conventional drugs may induce ferroptosis in cancer cells such as Sulfasalazine, Artesunate, Temozolomide, and Cisplatin (26). Herein, we predicted the small molecular drugs targeting ferroptosis. For example, we found that mTOR (LY-294002 and sirolimus) and PI3K inhibitors (wortmannin) could be potential ferroptosis inducers. Consistently, a preclinical study has reported that PI3K-AKT-mTOR pathway inhibits ferroptosis and inhibition of PI3K and mTOR can activate ferroptosis in cancer cells (27). Combination of ferroptosis inducers and chemotherapeutic drugs exhibits remarkably synergistic effects on anti-cancer activities (28). Here, we found that ferroptosis-related molecular subtypes were markedly related to the sensitivity to A.443654, AZD.0530, AZD6482, AZD7762, AZD8055, BAY.61.3606, Bicalutamide, and CGP.60474. It appeared that patients in C1 had higher sensitivity to these chemotherapeutic drugs. Among them, AZD6482 is an inhibitor of PI3K that is related to ferroptosis (29). AZD7762 can overcome cisplatin resistance in ovarian cancer (30). AZD8055, an inhibitor of mTORC1/2, can strengthen the sensitivity to MEK inhibitor Trametinib in ovarian cancer cells (31).

We constructed a 15-ferroptosis mRNA signature for predicting the prognosis of ovarian cancer, composed of CDKN1B, CXCR4, FAS, FOS, FOXO1, GABARAPL1, HDAC1, IFNG, IL24, MTMR14, NFKB1, PEX3, PPP1R15A, RB1, and SIRT2. All of them could be involved in the progression of ovarian cancer. For example, low CDKN1B expression is indicative of poor prognosis for ovarian cancer (32). CXCR4 is a critical determinant for tumor initiation, progression as well as metastasis of ovarian cancer (33). The intuitive and effective nomogram combining the signature and age was developed, which could be expediently employed for predicting patients' prognosis. These mRNAs could become potential therapeutic targets for ovarian cancer treatment.

Nevertheless, several limitations should be pointed out. Firstly, this was a retrospective study. The subtypes and signature models should be validated in a larger and multi-center ovarian cancer cohort. Secondly, the roles of ferroptosis-related mRNA in ovarian cancer required to be investigated in more experiments.

## Conclusion

Collectively, this study characterized two ferroptosis-related molecular subtypes in ovarian cancer, with distinct prognosis, tumor microenvironment and sensitivity to chemotherapeutic drugs. We predicted several underlying small molecular drugs against ovarian cancer such as LY-294002, sirolimus and wortmannin. A 15-ferroptosis mRNA signature was constructed, which robustly predicted the outcomes. These mRNAs could be promising therapeutic targets. Following combining this signature and age, we established an intuitive and effective nomogram, which could be applied for assisting precision treatment. Taken together, our findings indicated that inducing ferroptosis could be a promising therapeutic approach for ovarian cancer.

## Data Availability Statement

The datasets presented in this study can be found in online repositories. The names of the repository/repositories and accession number(s) can be found in the article/Supplementary Material.

## Ethics Statement

The studies involving human participants were reviewed and approved by the Ethics Committee of Cangzhou Central Hospital (2019045). The patients/participants provided their written informed consent to participate in this study.

## Author Contributions

SZ conceived and designed the study. JZ conducted most of the experiments, data analysis, and wrote the manuscript. JX and PH participated in collecting data and helped to draft the manuscript. All authors reviewed and approved the manuscript.

## Conflict of Interest

The authors declare that the research was conducted in the absence of any commercial or financial relationships that could be construed as a potential conflict of interest.

## Supplementary Material

Supplementary Table 1 A list of ferroptosis-related mRNAs.

Supplementary Table 2 25 prognosis-related ferroptosis mRNAs in ovarian cancer.

## Abbreviations

OS: overall survival
TCGA: The Cancer Genome Atlas
GEO: Gene Expression Omnibus
CDF: cumulative distribution function
PCA: principal components analysis
ESTIMATE: Estimation of stromal and immune cells in malignant tumors using expression data
CMap: Connectivity map
DEGs: differentially expressed genes
MoA: mode-of-action
ssGSEA: single sample gene set enrichment analysis
GDSC: Genomics of Drug Sensitivity in Cancer
IC50: half maximal inhibitory concentration
LASSO: least absolute shrinkage and selection operation
GEPIA: Gene Expression Profiling Interactive Analysis
ROCs: receiver operating characteristic curves
HR: hazard ratio
CI: confidence interval.
